# Curved Microneedle Array-Based sEMG Electrode for Robust Long-Term Measurements and High Selectivity

**DOI:** 10.3390/s150716265

**Published:** 2015-07-06

**Authors:** Minjae Kim, Taewan Kim, Dong Sung Kim, Wan Kyun Chung

**Affiliations:** Department of Mechanical Engineering, Pohang University of Science and Technology, Pohang 790-784, Korea; E-Mails: minjaekim@postech.ac.kr (M.K.); ktw2020@postech.ac.kr (T.K.); smkds@postech.ac.kr (D.S.K.)

**Keywords:** surface electromyography, microneedle array, curved electrode

## Abstract

Surface electromyography is widely used in many fields to infer human intention. However, conventional electrodes are not appropriate for long-term measurements and are easily influenced by the environment, so the range of applications of sEMG is limited. In this paper, we propose a flexible band-integrated, curved microneedle array electrode for robust long-term measurements, high selectivity, and easy applicability. Signal quality, in terms of long-term usability and sensitivity to perspiration, was investigated. Its motion-discriminating performance was also evaluated. The results show that the proposed electrode is robust to perspiration and can maintain a high-quality measuring ability for over 8 h. The proposed electrode also has high selectivity for motion compared with a commercial wet electrode and dry electrode.

## 1. Introduction

During a muscle contraction, motor unit action potentials are generated and propagated up to the skin. A surface electromyography (sEMG) signal is the measured muscle activation signal at the skin surface. However, such sEMG signals are noisy and complex due to the stochastic nature of the signal [1].

The most commonly-used electrode is the wet type, which includes an electrolyte to decrease skin impedance by diffusing into the skin layer. However, this type of electrode is not suitable for long-term measurements, because the electrolyte tends to dry out and is easily influenced by skin conditions, such as sweat accumulation. Sweat distorts the signal and degrades the skin contact conditions [2]. Flexible polymer-based dry electrodes [3–5] have been proposed to improve skin contact conditions. However, the signal quality is still limited, because the skin impedance remains high, and the effects of environmental interference are not negligible [6]. Both types of electrodes restrict the measurement conditions and have hampered the expansion of sEMG-based applications [7].

For example, sEMG measurements using these conventional electrodes are not appropriate in dynamic conditions, such as running and intensive exercise. Perspiration and intense motion degrade the contact conditions of the electrode and cause signal distortion. Furthermore, the limitations of the electrodes make them difficult to use in applications that require long-term usage. For example, a prosthetic hand, one application of sEMG, requires over 8 h of usage [8]. Thus, there is a continuing need for the development of new types of electrodes.

One solution to these problems is a microneedle array (MNA) electrode that penetrates the highly resistive outermost skin layer of dead skin and makes direct contact with the living inner skin layer. Although MNA electrodes have been proposed previously, these reports focused on feasibility and did not consider human factors, such as safety and stable skin contact. Photolithography-based electrodes [9–11] showed feasibility, but these silicon-based electrodes were easily broken due to the brittleness of silicon. Broken silicon needles can cause skin irritation, infection, and pain. To alleviate these issues, biocompatible polymer-based MNA electrodes were fabricated using UV exposure [12]. However, only skin impedance and the signal-to-noise ratio (SNR) were evaluated. Furthermore, most of the conventional MNA electrodes have a rigid planar substrate, which cannot compensate for skin curvature.

We developed a curved MNA electrode made of a biodegradable polymer, polylactic acid (PLA). The curved substrate maintains stable and comfortable skin contact. The proposed electrode is attached to the skin with a flexible band to fix the electrode to the skin. Using the proposed MNA electrode, we evaluated its performance in terms of long-term usability and insensitivity to perspiration. We also performed a discrete motion prediction task to evaluate motion selectivity and long-term usability. The results demonstrate that the proposed MNA electrode achieves better performance than conventional electrodes.

The structure of the skin and simplified electrical circuit models for electrodes are described in Section 2. The fabrication process of the curved MNA electrode with the band is described in Section 3, and the experimental results to evaluate its signal quality are presented in Section 4. The motion discriminating performance is discussed in Section 5.

## 2. Skin Structure and Simplified Electrical Circuit Models

### 2.1. Skin Structure

**Figure 1:**
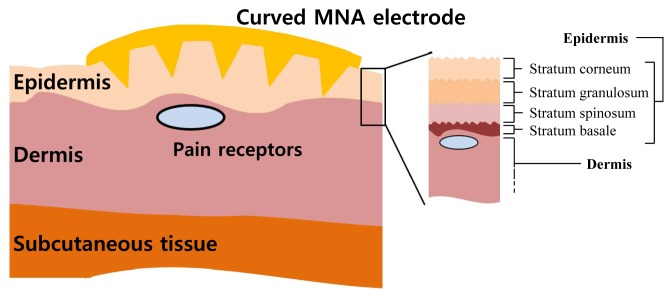
Structure of the skin with the microneedle array (MNA) electrode.

The skin has multiple layers: an epidermis, a dermis and a subcutaneous tissue (Figure 1). The outer skin layer, the epidermis, consists of a stratum corneum (SC), a stratum granulosum, a stratum spinosum and a stratum basale. The thickness of the epidermis varies considerably between different regions of the body. Cells of the epidermis are produced in the inner layers and migrate to the outer layers. The SC is 10–20 µm thick and is composed of dead cells that protect the underlying tissues. The SC has very high impedance because of its barrier properties. Other layers of the epidermis are composed of living cells that are electrically conductive. The dermis contains blood vessels and nerves with pain receptors. If any object touches the dermis, pain receptors detect the object, and the human brain perceives pain. The goal of the MNA electrode is to penetrate the SC layer and directly contact the living epidermis layer without touching the dermis layer.

### 2.2. Simplified Electrical Circuit Models

**Figure 2:**
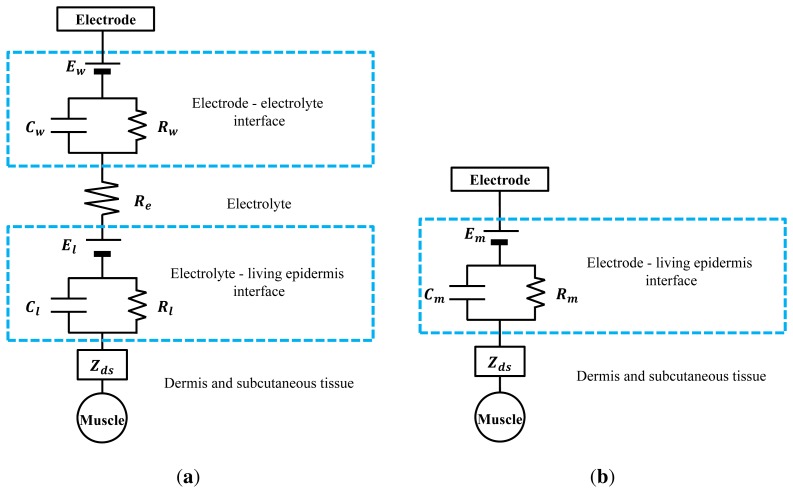
Electrical equivalent circuit models of the standard wet and MNA electrodes. (**a**) Wet electrode model; (**b**) MNA electrode model.

When the electrode contacts the skin, ionic currents of muscle signals are converted into electric currents in the electrodes. The paths of these currents can be expressed as electrical equivalent circuit models (Figure 2).

In the case of the wet electrode in Figure 2a, the impedance between the electrode and the electrolyte can be modeled as a combination of capacitance, resistance and half-cell potential [13]. These elements describe the polarization effect, caused by the ionic differences between the electrode and the electrolyte. Furthermore, the ionic concentration difference between the electrolyte and the living epidermis causes additional impedance. The dermis and the subcutaneous tissue can be modeled as impedances. The equivalent impedance of the wet electrode can be modeled as:

(1) $$\left|Z_{\text{Wet}}\right|=\left|Z_{ds}\right|+\frac{R_{l}}{\sqrt{1+{\left(\omega R_{l}C_{l}\right)}^{2}}}+R_{e}+\frac{R_{w}}{\sqrt{1+{\left(\omega R_{w}C_{w}\right)}^{2}}}$$

where *Z_ds_* indicates the combined impedance of the dermis and the subcutaneous tissue and *R_e_* indicates the resistance of the electrolyte. The combination of *R_l_* and *C_l_* is the electrolyte to living epidermis interface impedance, and the combination of *R_w_* and *C_w_* corresponds to the electrode to electrolyte interface impedance. All elements, apart from *Z_ds_*, depend on the condition of the electrolyte. As noted, the electrolyte dries over time and is readily influenced by environmental factors, such as skin condition and humidity. Because the amplitude of noise depends on the impedance [14], the consistency of the SNR is not guaranteed when wet electrodes are used.

In the case of the MNA electrode as shown in Figure 1, the electrical circuit model is simple (Figure 2b). The MNA electrode contacts the living epidermis directly, with no conductive medium. The polarization effect is caused only by the electrode and the living epidermis interface. The conditions of the electrode and skin do not change significantly, so the elements remain within a certain range of values. Thus, the MNA electrode can achieve low impedance and large SNR over time. The equivalent impedance of the MNA electrode can be modeled as:

(2) $$\left|Z_{\text{MNA}}\right|=\left|Z_{ds}\right|+\frac{R_{m}}{\sqrt{1+{\left(\omega R_{m}C_{m}\right)}^{2}}}$$

where the combination of *R_m_* and *C_m_* indicates the electrode to living epidermis interface impedance.

If the electrolyte property of the wet electrode is identical to the epidermis property, ideally, *R_l_* = *C_l_* = 0, and then, the following equation is obtained:

(3) $$\frac{R_{w}}{\sqrt{1+{\left(\omega R_{w}C_{w}\right)}^{2}}}=\frac{R_{m}}{\sqrt{1+{\left(\omega R_{m}C_{m}\right)}^{2}}}$$

Then, we can show that following property holds:

(4) $$\left|Z_{\text{Wet}}\right|\approx \left|Z_{\text{MNA}}\right|+R_{e}$$

Thus, the impedance of the MNA electrode is always lower than that of the wet electrode.

## 3. Fabrication

In this section, the fabrication processes of the curved MNA electrode and the flexible band are described. Briefly, the PLA-based MNA was fabricated using a polydimethylsiloxane (PDMS) replica molding. Then, the MNA was annealed on the curved substrate above its glass temperature. Next, biocompatible silver/titanium layers were coated, and wires were connected. While the electrode was attached to the skin, a flexible band wrapped the electrode and fixed it.

### 3.1. Microneedle Array

**Figure 3:**
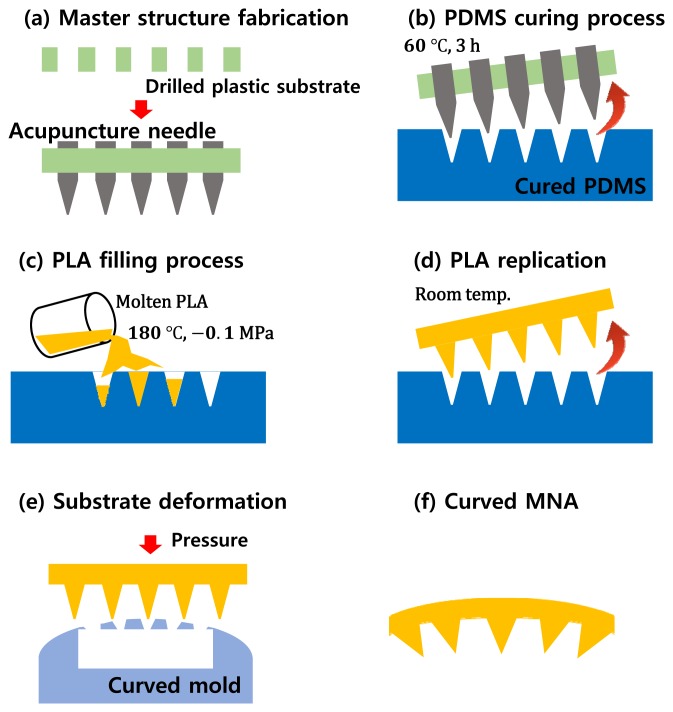
Fabrication process for the microneedle array. (**a**) Master structure fabrication with acupuncture needles; (**b**) PDMS mold fabrication using the master structure; (**c**) molten PLA filling process in a vacuum; (**d**) microneedle array replication; (**e**) substrate deformation on the curved mold; (**f**) fabricated curved microneedle array.

**Figure 4:**
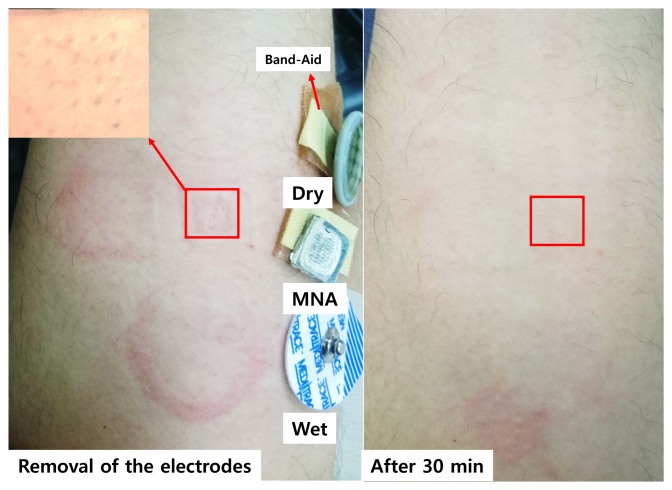
Examination of skin of the forearm wearing the electrodes for 8 h.

The MNA was fabricated using commercially available acupuncture needles. Our detailed MNA fabrication process is described in [15,16]. A master template with acupuncture needles was prepared. Then, the MNA was fabricated by a micro-molding process with a PDMS mold to replicate the master template. Here, PLA, a biodegradable polymer was used for the replication as an MNA substrate because the PLA has a moderate melting/glass temperature for the replication. Furthermore, although the PLA can be degraded on the skin, the silver/titanium coating prevents such degradation during use. This fabrication process is described in Figure 3. The fabricated MNA electrode consisted of a 10 by 10 needle array with a pitch of 0.95 mm at the base of 8.5 by 8.5 mm^2^. The diameters of the tip and the body of each needle were 10 and 150 µm, respectively. The length of each needle was about 350 µm, which was determined to measure the sEMG signal without causing pain. Furthermore, no itching or inflammation was observed during and after wearing for 8 h, as shown in Figure 4.

### 3.2. Curved Microneedle Array Electrode

**Figure 5:**
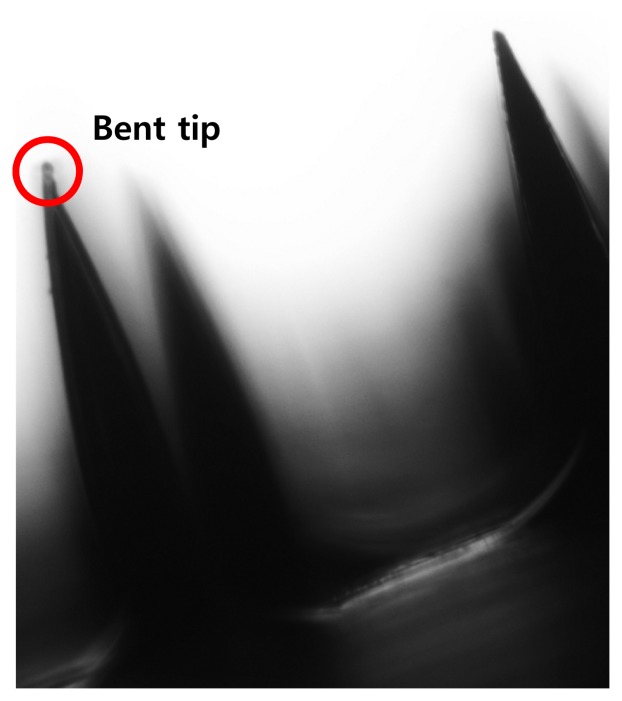
Needle tip condition after the annealing process.

**Figure 6:**
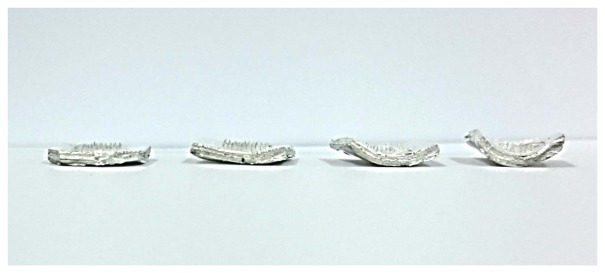
Various MNA electrodes having different curvatures.

The ideal shape for a sEMG electrode is a curved shape to compensate for the skin's curvature and to ensure comfortable skin contact. After the MNA was fabricated, the MNA was annealed on a curved substrate above its glass temperature. The curved substrate was prepared using commercial clay, the shape of which can be modified easily. Figure 5 describes the microscopic image of the needle tip after the annealing process. Although several needles showed bending, the effect of the tip bending can be negligible. After the annealing process, the silver/titanium layers were added using an e-beam evaporator, and wires were connected to conventional amplifiers. While the electrode was attached to the skin, a flexible band wrapped the electrode's location. The curved substrates with different curvatures are shown in Figure 6.

### 3.3. Band System

**Figure 7:**
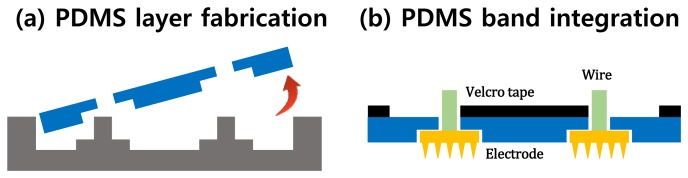
Fabrication process for the PDMS band array. (**a**) Base PDMS layer fabrication; (**b**) the MNA electrode integration and Velcro tape attachment.

**Figure 8:**
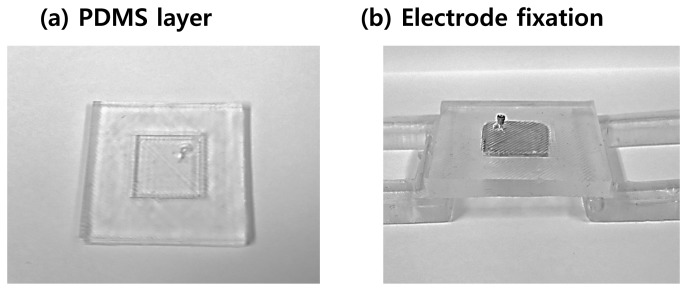
Images of the PDMS layer for a single electrode. The thickness of the band depends on the electrode thickness. (**a**) PDMS layer without Velcro tape; (**b**) PDMS layer with the fixed MNA electrode.

**Figure 9:**
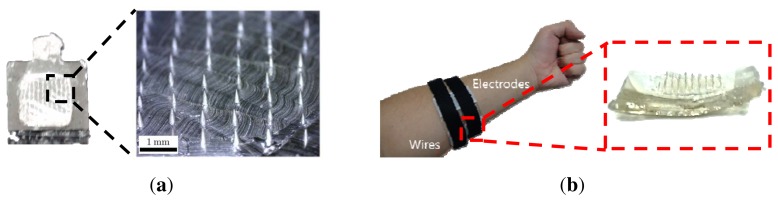
Images of the proposed MNA electrode and band system. (**a**) Microscopic image of the planar MNA electrode; (**b**) image of skin-attached PDMS band.

PDMS is an appropriate material for a wearable band. The flexibility of PDMS allows the system to compensate for skin deformation, and the biocompatibility of PDMS ensures that it does not cause adverse effects to the user, even in long-term use. The fabrication process is described in Figure 7. A thin PDMS layer, which has holes fitted to the MNA electrode, was fabricated as shown in Figure 7a. The number of holes depends on the configuration of the sEMG channels. Then, the fabricated MNA electrodes were fixed to the PDMS layer, and one side of the Velcro tape was attached to the PDMS layer (Figure 7b). Finally, the PDMS layer was attached to the skin and fixed using the other side of the Velcro tape. The fabricated MNA electrode and the band system are shown in Figures 8 and 9.

## 4. Signal Quality Evaluation

We consider insensitivity to environmental interference and long-term usability as important factors for the evaluation of sEMG electrodes. In this section, signals were evaluated by comparing the proposed electrode with a commercial wet electrode (Medi-trace 100, Covidien, Mansfield, MA, USA) and a dry electrode (Drypad electrode, Cognionics, San Diego, CA, USA).

The measured sEMG signals were amplified by a factor of 1000 with an amplifier (EMG-KIT, PhysioLab, Busan, Korea) and were sampled digitally at 1 kHz using a data acquisition board (NI-DAQ 6259, National Instruments, Austin, TX, USA). One subject participated, and the signals were measured five times per each type of electrode.

### 4.1. Sensitivity to Perspiration

**Figure 10:**
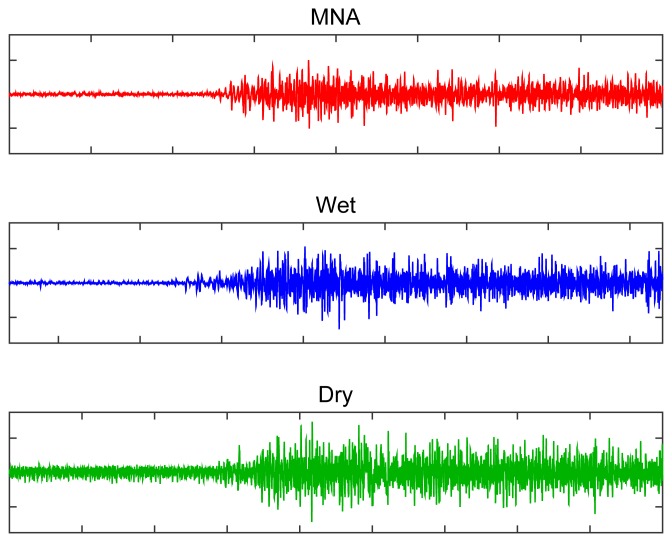
Measured sEMG signal during the maximum voluntary contraction from the FCR before the exercise. Signals were measured independently.

Sweat is released from the glands in the dermis and supplies an additional route for the sEMG signal. Sweat reduces contact stability, distorts the signal and reduces signal amplitude during isometric contraction. To investigate the sensitivity to perspiration, the proposed MNA electrodes were attached to the left forearm flexor carpi radialis (FCR) with commercial wet and dry electrodes. Signals from the electrodes were measured during a maximum voluntary contraction before and after 20 min of stair-climbing exercise. Because the electrode locations were not the same, the measured RMS amplitudes were normalized to compare the tendencies. Raw signals are described in Figure 10.

**Figure 11:**
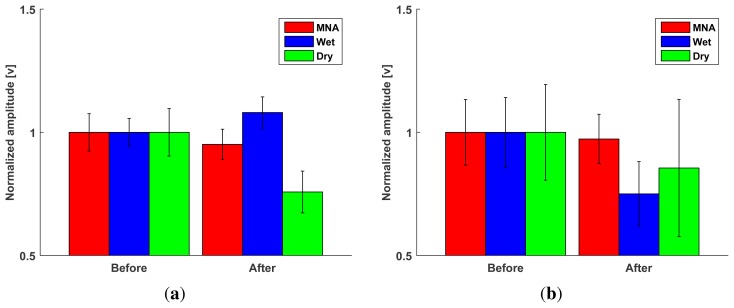
Performance evaluation of the proposed MNA electrode compared with the commercial electrodes in terms of insensitivity to perspiration before and after the exercise. Bars: ±1 SD, *n* = 5. (**a**) Changes in the baseline RMS amplitudes in relaxed state; (**b**) changes in the RMS amplitude during the maximum voluntary contraction.

The MNA electrode showed less variation than the wet and dry electrodes before and after exercise (Figure 11). The RMS amplitude of not only the dry electrode, but also the MNA electrode in the rest condition decreased after exercise, because the sweat improved the contact between the electrode and the skin. In the case of the MNA electrode, there was no significant difference (*p* = 0.19). In contrast, the RMS amplitude of the wet electrode in the rest condition increased (*p* = 0.04) because the sweat contaminated the electrolyte and decreased its contact stability. Furthermore, both commercial electrodes showed a significant amplitude reduction during maximum voluntary contraction due to sweating. However, the MNA electrode showed an insignificant amplitude decrease, and the amplitude variation was less (*p* = 0.53). These results indicate that the MNA electrode was relatively insensitive to the effects of sweating.

### 4.2. Long-Term Usability

Long-term usability can be evaluated by the temporal change in baseline amplitude, which is the signal amplitude in the relaxed state. High baseline amplitude increases the threshold of the onset of the muscle activation, making it more difficult to analyze the sEMG signal. To evaluate long-term usability, the proposed MNA electrode and the commercial electrodes were attached to the extensor carpi radialis (ECR) location; then, the baseline amplitude was measured for 8 h. The measured signals were normalized to compare the tendencies.

**Figure 12:**
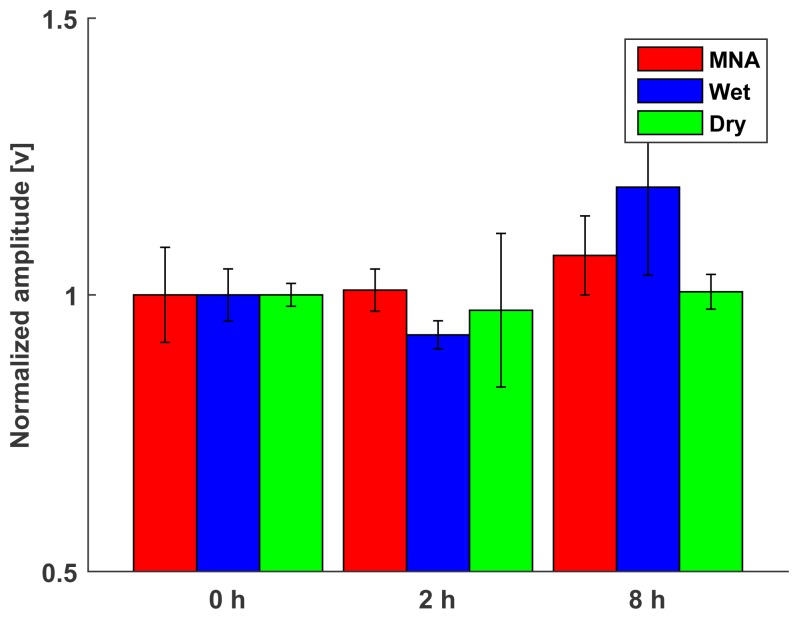
Temporal changes in the baseline RMS amplitudes. Bars: ±1 SD, *n* = 5.

After 2 h, the baseline RMS amplitude of the wet electrode decreased due to the electrolyte settling, because the wet electrode requires time to allow the electrolyte to diffuse. After that, the amplitude tended to increase as time passed due to drying of the electrolyte. Therefore, only data from 0 h, 2 h, and 8 h are described in Figure 12. In contrast, the MNA electrode and the dry electrode showed similar baseline RMS amplitudes, because both types of electrodes contact the skin with no medium. The results indicate that the temporal changes in signal quality were not significant for the MNA electrode and dry electrode (the *p*-values were 0.24 and 0.73 , respectively), while the wet electrode was affected considerably (*p* < 0.05).

## 5. Motion-Discriminating Performance Evaluation

In this section, the motion discriminating performance evaluation is described. The measured sEMG signals were amplified by a factor of 1000 with an amplifier (PSL-iEMG, PhysioLab, Korea) and were sampled digitally at 2 kHz using a data acquisition board (NI-DAQ 6259). Then, a digital low-pass filter with a 490-Hz cut-off frequency and a digital high-pass filter with a 10-Hz cut-off frequency were applied. In both experiments, linear discriminant analysis (LDA)-based classification was performed. For feature extraction, the sEMG signals obtained were divided into 200-ms windows, and the increment was 50 ms. The sEMG signals in each window were converted to feature vectors that contained the mean absolute value (MAV), wavelength (WL) and fourth-order auto-regressive coefficients (AR), as described in Table 1. Then, the overall features were divided into two groups: learning and testing for classification, for which the ratio was 7:3. Then, the LDA and Mahalanobis distance-based classification was performed. To evaluate the algorithm, trials were performed 1000 times for randomly-divided feature sets.

### 5.1. Selectivity Evaluation

**Figure 13:**
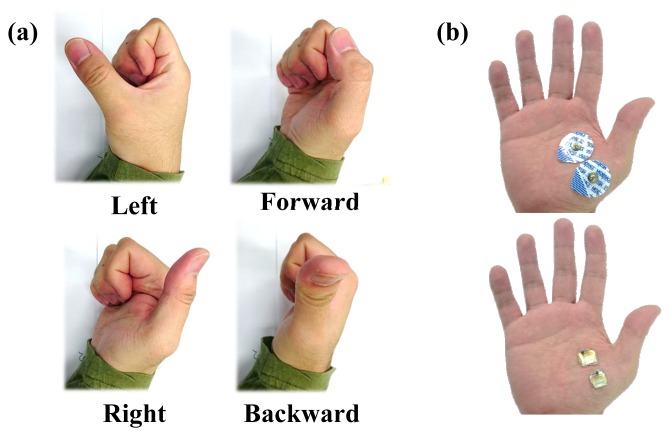
(**a**) Configurations of the thumb that were used in motion classification; (**b**) electrode placement.

To investigate the selectivity of the electrode, a thumb motion classification was performed. The thumb has a universal joint, so it can represent various motions (Figure 13a). The thumb has a complex muscle group, located in the highly deformable palm. Thus, the electrodes for thumb motion classification require stable contact and high selectivity of motion. The performance was compared with those of the commercial wet electrode and the dry electrode. Five subjects participated in this experiment.

#### 5.1.1. Data Processing

Two electrodes were attached to the palm, where muscle contractions are performed according to the thumb motion without anatomical considerations, as shown in Figure 13b. The reference electrode was attached to the elbow. During sEMG signal collection, the target motion was shown on a computer screen for 4 s. At the same time, the subject performed the corresponding motion with a comfortable force to avoid muscle fatigue. The subject had 2 s to relax the muscle before the next motion. Each motion was repeated three times. From the 4 s of each gesture, the first 0.5 s and the last 0.5 s were discarded, as the subject had that time to prepare the motion.

#### 5.1.2. Results

**Figure 14:**
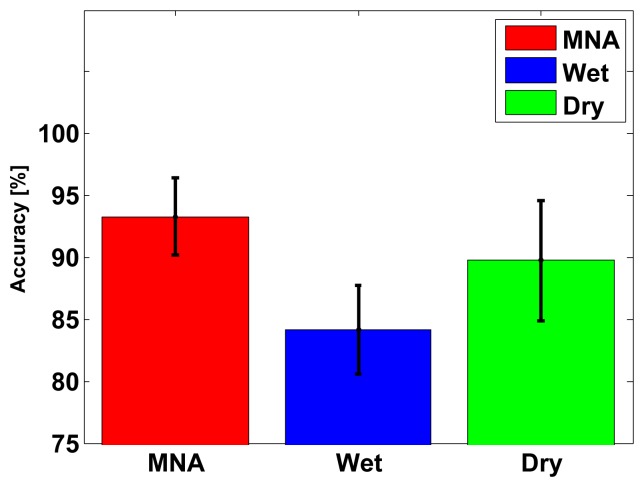
Mean accuracy and standard deviation of the classification accuracy using three types of electrodes (Subject 1). The accuracy of the proposed MNA electrode was significantly higher.

**Figure 15:**
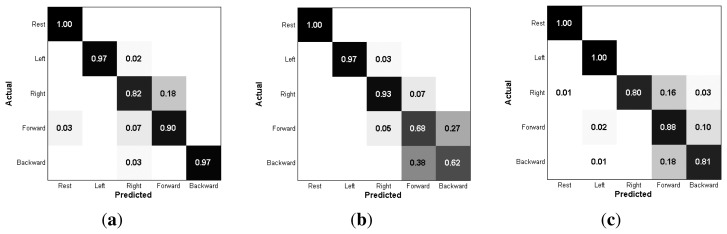
Confusion matrix of three types of electrodes (Subject 1). The commercial wet and dry electrodes showed higher error rates between motions, which means that the proposed MNA electrode has a higher selectivity of motions. (**a**) Proposed MNA electrode; (**b**) commercial wet electrode; (**c**) commercial dry electrode.

**Figure 16:**
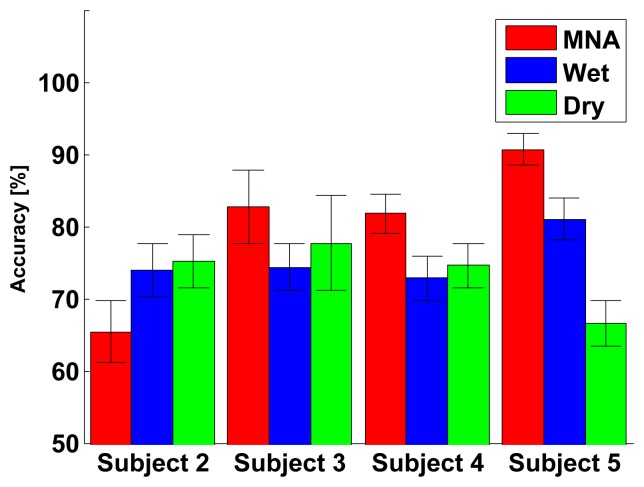
Classification accuracies for Subject 2 to Subject 5. In the case of Subject 2, the MNA electrode showed lower accuracy. However, other subjects showed a similar tendency.

The classification accuracy distribution is described in Figure 14 and Table 2. The accuracy of the MNA electrode was significantly higher than that of the commercial electrodes, where the accuracy was defined as average, best or worst. Thus, the proposed electrode achieved higher discriminating performance for a complex muscle group. Although the electrode placement and the size of both electrodes were similar, the wet electrode produced more crosstalk noise, because the diffused electrolyte increased the effects of the activation of nearby muscles. As described in the confusion matrix (Figure 15b), the wet electrode showed a higher error rate between forward motion and backward motion, indicating that the measured signals in both cases were difficult to distinguish. Similarly, the dry electrode measured signals from the skin surface only, so the dry electrode produced more crosstalk noise than the MNA electrode (Figure 15c). However, the proposed electrode showed a higher true rate, as shown in Figure 15a. The one-way analysis of variance showed that there was a significant difference (*p* < 0.001). The selectivity depends on the electrode contact conditions, so the proposed electrode achieved higher muscle selectivity, because the tiny needles contacted locally with the conductive tissue in the inner skin layers with no medium. Other subjects showed a similar tendency, except Subject 2, as described in Figure 16.

### 5.2. Long-Term Classification Accuracy Evaluation

In this experiment, the relationship between the attachment time and the motion classification accuracy was investigated. Although many self-enhancing classification methods have been proposed, we focused only on the accuracy based on the test and learning data from the same session, because the highest classification accuracy can be achieved from data pairs from the same session [17]. The experiments were performed five times overall per electrode type, and then the accuracies were averaged.

#### 5.2.1. Data Processing

The experiment included six classes of wrist and hand motions: relaxed state, wrist extension, wrist flexion and rock-paper-scissors (hand close, hand open and two-finger extension). Two pairs of the electrodes were attached to the FCR and ECR. From the attachment, LDA classification was performed every 2 h for 8 h. The next day, the electrodes were placed in the same location, where the sites had been marked with a skin-friendly pen.

#### 5.2.2. Results

**Figure 17:**
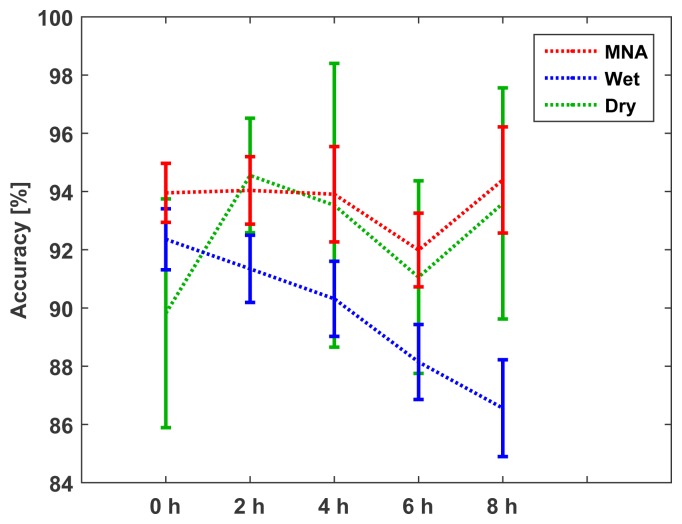
Temporal changes of the LDA classification accuracy.

The classification accuracy distribution is described in Figure 17. At the beginning of the attachment (0 h), the accuracy of the proposed electrode was slightly higher than that of the standard wet electrode. However, as time passed, the classification accuracy using the standard wet electrode decreased continuously, because the electrolyte was contaminated and dried out. With the proposed electrode, the signal quality depended only on the contact conditions, so the classification accuracy remained high. Although the dry electrode maintained high accuracies during the attachment, it showed a large variance, because the flat surface did not guarantee stable skin contact during the measurement.

## 6. Conclusions

In this paper, a curved microneedle array electrode with a PDMS band was described. The tiny microneedles penetrate the highly-resistive dead epidermis and make direct contact with the living epidermis, so the microneedle array electrode can provide low impedance signals without an electrolyte. The curved shape compensates for the skin's curvature, and the PDMS band maintains stable skin contact during the measurements. The stable contact increases the stability of signal measurement and reduces the effects of environmental interference, such as perspiration. The proposed electrode can also be used for long-term measurements. Another important advantage of the proposed electrode is its high selectivity. The motion classification evaluation indicated that although the electrode area of the proposed electrode was similar to that of the commercial electrodes, the proposed electrode had higher selectivity.

## Figures and Tables

**Table 1:** Features extracted from each sEMG signal. WL, wavelength; AR, auto-regressive coefficient.

**Acronym**	**Formula**
MAV	$\frac{1}{L}\sum_{i=1}^{L}\left|x_{i}\right|$
WL	$\sum_{L}^{i=1}\left|\Delta x_{i}\right|$
AR	[*α*], where $x_{i}=\sum_{r=1}^{4}\left|\alpha_{r}x_{i-r}+{ɛ}_{i}\right|$

**Table 2:** Thumb motion classification accuracy (Subject 1).

**Method**	**Test Accuracy (Percentage)**		

	**Average**	**Best**	**Worst**
Proposed MNA electrode	93.3	100	81.7
Wet electrode	84.2	95.0	73.3
Dry electrode	89.7	98.6	70.3
